# A new species of *Angelopteromyia* Korneyev, 2001 (Diptera, Platystomatidae) from Iran, with the key to the species

**DOI:** 10.3897/zookeys.224.3795

**Published:** 2012-09-29

**Authors:** Saeed Mohamadzade Namin, Jamasb Nozari

**Affiliations:** 1 Department of Plant Protection, Faculty of Agriculture, Varamin-Pishva Branch, Islamic Azad University, Varamin, Iran; 2 Department of Plant Protection, Faculty of Agriculture, University of Tehran, Karaj, Iran

**Keywords:** Diptera, Platystomatidae, Iran, new species

## Abstract

*Angelopteromyia korneyevi* Mohamadzade Namin, **sp. n.** from Iran is described and figured. The new species is similar to other species of *Angelopteromyia* in having abdominal spiracles 5 of females not approximated medially, as well as clypeus extended postero-ventrally, antenna shorter than face, and R_1_ and R_4+5_ setulose on dorsal side. It differs from other species of *Angelopteromyia* by having mostly brown wing with 3 hyaline crossbands and a few hyaline spots, and dark brownish basal and costal cells without hyaline spots.

## Introduction

The signal flies (Platystomatidae) are small to large-sized flies (3.5–20 mm) often with grayish microtrichose or bright and metallic blue or green body. Wings are usually strongly patterned and the cell bcu is closed by arcuate or straight vein at apex, without a posteroapical lobe (Korneyev 2001). There are about 1200 described species in 119 genera worldwide (McAlpine 2001), with about 66 species in 8 genera occurring in the Palaearctic Region (Soós 1984, Korneyev 2001). Keys to the species of the family Platystomatidae was provided by Hennig (1945); that paper is partly out-of-date now; the most comprehensive keys to the Palaearctic genera of the family were provided by McAlpine (1998) and Korneyev (2001).

*Angelopteromyia* Korneyev, 2001 is a genus of the subfamily Platystomatinae with 3 described species occurring in Central Asia (*Angelopteromyia alf* Korneyev, 2001, *Angelopteromyia chvalai* Korneyev, 2001 and *Angelopteromyia merzi* Korneyev, 2001). According to Korneyev (2001), *Angelopteromyia* differs from all other Platystomatidae genera by the following combination of characters: facialia wider than parafacialia, projected anterolaterally; clypeus extended postero-ventrally; antenna shorter than face; 1 medial and 1 lateral vertical, 0–1 genal setae. Thorax slightly wider than head, with 1 postpronotal, 0–2+1–6 supraalar, 1–2 postalar, 1–2 intraalar, 1–2 pairs of Scapular (usually inconspicuous), 1–2 dorsocentral close to scutellum, 1–2 Acrostichal, 1+1 notopleural, 1 anepisternal, 0 katepisternal, 3–5 pairs of scut; wings with dark reticulate pattern; cells bcu and bm closed by straight or arcuate vein, R_1_ and R_4+5_ setulose on dorsal side; female abdominal spiracles 5 not approximated medially; epandrium long, surstyli arisen posteriorly with 1 claw-like prensiseta, the second prensiseta is not clearly visible; phallus with 2 long mostly separated acrophallic tubes; hypandrium asymmetrical anteriorly; vanes of phallapodeme separate. It clearly differs from *Platystoma*, which possesses also a reticulate wing pattern, by the lateral position of abdominal spiracles 5 of female (strongly approximated ventrally in *Platystoma*).

While studying the tephritoid flies fauna in West Azerbaijan Province (Iran), a previously undescribed species of *Angelopteromyia* was collected by the first author. The new species is described and figured below.

## Methods

Material is collected by standard sweeping net and minuten-pinned in side. Morphological terminology generally follows McAlpine (1981). The material examined is deposited in collections of the following institutions:

JAZM Jalal Afashar Zoological Museum, College of Agriculture, University of Tehran, Karaj, Iran.

SIZK I. I. Schmalhausen Institute of Zoology, National Academy of Sciences of Ukraine, Kiev, Ukraine.

## Results

### Angelopteromyia korneyevi

Mohamadzade Namin sp. n.

urn:lsid:zoobank.org:act:CDE4B3D3-9BBB-45FC-A9F7-A71B0C00394B

http://species-id.net/wiki/Angelopteromyia_korneyevi

Figs 1 [Fig F2] –12

#### Type material.

Holotype m#: Iran, West Azerbaijan Province, 10km west Ziveh, 37°08'N, 44°52'E, h 2700m, 8 July 2011 (Mohamadzade leg.) (JAZM).

Paratypes: 4♂, 1♀, same collection data as in holotype (JAZM; SIZK and first author’s personal collection).

#### Description.

**Male.**

Head (fig. 2): Head length: height: width ratio = 1: 1.26: 1.48. Eye elongate elliptical. Lunule, antennal grooves and facial ridge black. Frons black, densely dark brownish tomentose, with black setulae and with shining black dots at bases of setulae and setae; ocellar triangle black. Lower two thirds of occiput conspicuously expanded posteriorly; postocellar, occipital and supracervical setulae black. Antenna black, first flagellomere rounded apico-dorsally, arista brownish black and grayish microtrichose with small pubescence. Antennae short and shorter than face, pedicel about half as long as first flagellomere, apical part of first flagellomere rounded and grayish microtrichose. Face shining black, concave in profile. Clypeus large, subshining black. Gena subshining dark brown and 1.2 times as long as first flagellomere. Sides of frons near compound eyes with triangular white microtrichose area. Anterior part of postgena around posterior margin of compound eyes with white microtrichose area that reaches to posterior margin of head. Proboscis brownish black, labellum large and black with long black setae. Palp rounded at apex, black with long black setae. Chaetotaxy: 2 orbital, 1 ocellar, 1 medial vertical, 1.15 times as long as 1 lateral vertical and 1.3 times as long as orbital setae and about twice as long as ocellar seta. All setae and setulae black.

**Figures 1–6.**
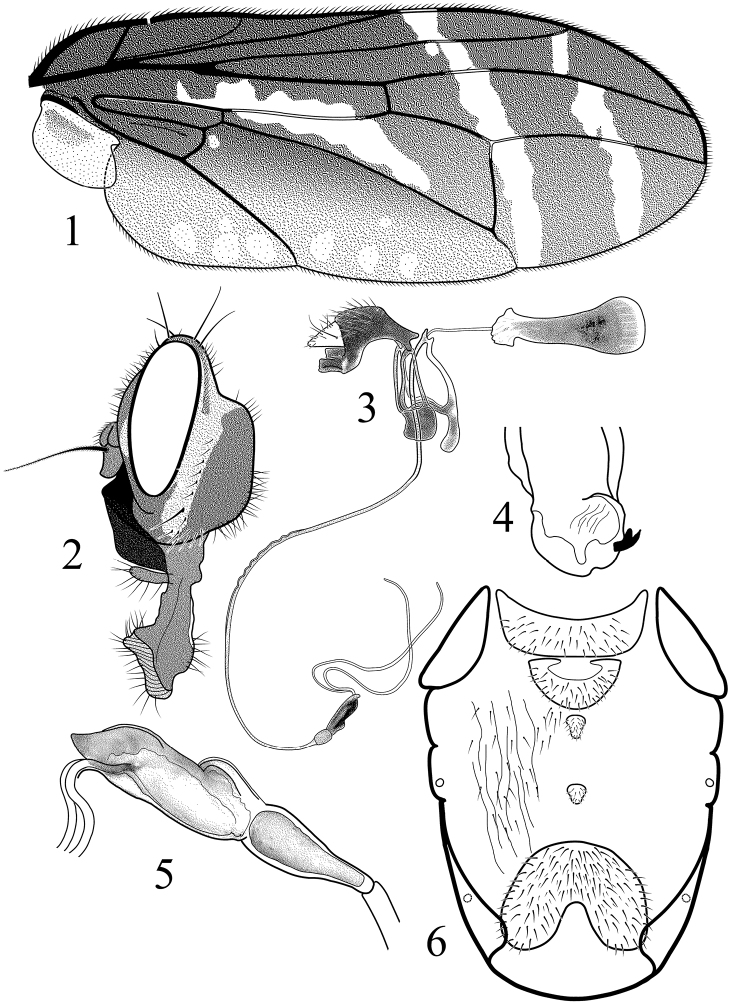
*Angelopteromyia korneyevi* sp. n. **1** wing **2** head in profile**3** male genitalia, right aspect **4** surstyli apex, ventral aspect **5** male terminalia **6** abdominal sclerites in male, ventral aspect.

Thorax: all parts of thorax subshining black with numerous black dots at bases of setulae and setae (fig. 12). Chaetotaxy: 1 postpronotal, 1+1 notopleural, 2+3 supra-alar, 1 intraalar, 1 dorsocentral, close to scutellum, 1 prescutellar acrostichal, 1 anepisternal and no katepisternal setae present. Scutellum black with 3 pairs of equal setae.

Wing (fig. 1) 2.4 times as long as wide, with dark brown disc, and pattern of 3 hyaline crossbands. Base of wing and costal cell dark brown. Pterostigma brown without hyaline spots. Only 2 oblique hyaline bands present in apical part of the wing: one band crossing wing from r_1_ cell to posterior margin of the wing. Another hyaline band crossing wing from r_2+3_ cell near terminal part of R_2+3_ to posterior margin of the wing. Posterior part of apical two-thirds of cell br with oblique hyaline crossband penetrating into cell dm. Anal lobe and cubital cell light brown, containing several hyaline spots at wing posterior margin. R_1_ and R_4+5_ setulose dorsally with 13–21 setulae (in holotype 13 on right and 15 on left wing) in whole length of R_4+5_. Penultimate section of M 2.3 times shorter than ultimate section and 1.2 times longer than dm-cu. Lower calypter light brown with dark brown spot in middle part. Knob of halter brown, stalk yellow.

Legs with black coxae, trochanters, femora and tibiae; fore tarsus black, only basal one-fourth of first and second tarsomeres yellow. First and second tarsomeres of mid and hind tarsus yellow, remaining tarsomeres black. Fore femur subshining black and sparsely microtrichose, with long black setulae and 2 rows of ventro-lateral long black setae. All tibiae and tarsi with black setulae (fig. 11).

Abdomen: subshining black, tergite 5 of male longer than tergites 1–4 together, with shining black posterior margin. Pleura velvet grey with black hairs. Sternites dark brown; sternite 5 of male very large and broad (fig. 6). Male terminalia as on figs 3–5. Proctiger triangular, swelling part of glans about three times longer than wide. Terminal filaments of acrophallus equally thick in whole length. Surstyli with one bifurcated claw-like prensiseta visible from ventral view (fig. 4).

Body length: 4.2 mm. Wing length: 4.2 mm

**Figures 7–10. F2:**
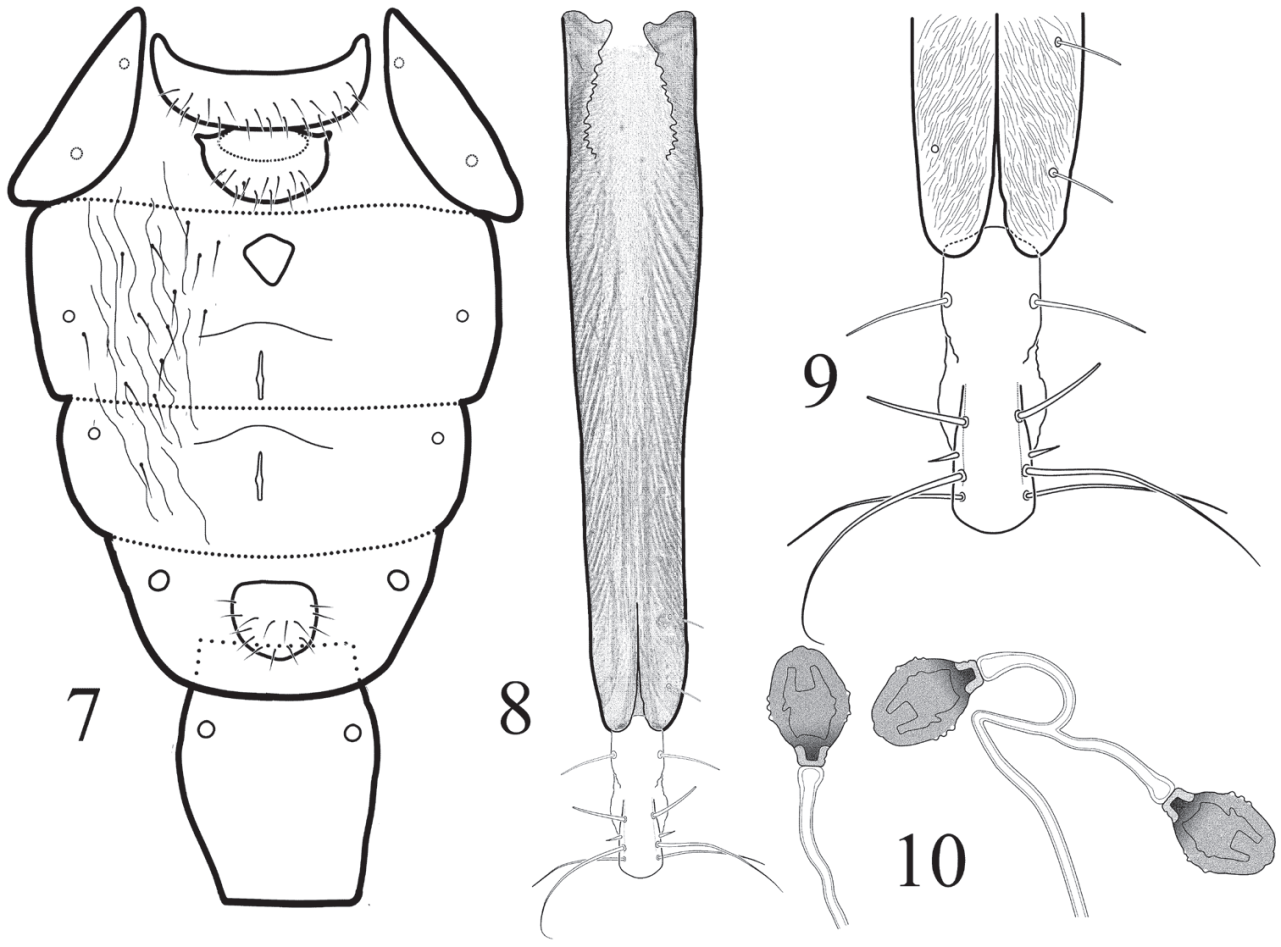
*Angelopteromyia korneyevi* sp. n. **7** abdominal sclerites in female, ventral aspect **8** aculeus, ventral aspect**9** aculeus apex, ventral aspect **10** spermathecae.

**Female.** Similar to male. Abdominal spiracles 5 of female not approximated medially (fig. 7). Female terminalia as on figs 8–9 and Spermatheca as in fig. 10.

**Measurements**. Male: Body length: 4.2–4.5 mm. Wing length: 4.0–4.7 mm; Female: 4.3 mm. Wing length: 4.0 mm

**Figures 11–12.**
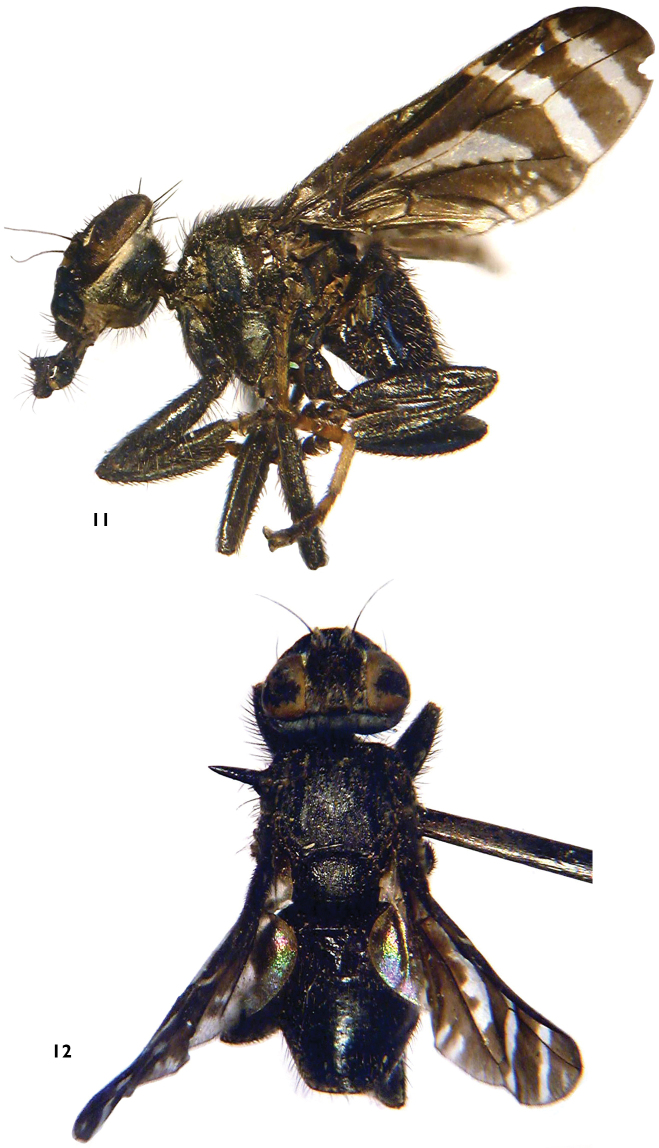
*Angelopteromyia korneyevi* sp. n. **11** ♂, total view, lateral **12** ♂, total view, dorsal.

#### Etymology.

The species is named for Dr. Valery A. Korneyev (I. I. Schmalhausen Institute of Zoology, Ukraine), who has made a valuable contribution to the study of Palaearctic Platystomatidae.

## Discussion

The new species is similar to other species of *Angelopteromyia* sharing most of generic characters, and first of all, abdominal spiracles 5 of females not approximated medially. *Angelopteromyia korneyevi* sp. n. differs from other species of *Angelopteromyia* in having entirely different wing pattern: dark brownish basal and costal cells without hyaline spots (in *Angelopteromyia alf*, *Angelopteromyia chvalai* and *Angelopteromyia merzi*, basal cells of wing reticulated with numerous hyaline spots). In addition, face in *Angelopteromyia korneyevi* sp. n. is completely shining black (only ventral half of face is shining black in *Angelopteromyia merzi* and half of face at least in the middle is densely microtrichose in *Angelopteromyia alf* and *Angelopteromyia chvalai* ventrally). Furthermore, the proctiger in *Angelopteromyia korneyevi* sp. n. is triangular in profile (apical part of proctiger is rounded in *Angelopteromyia alf* and *Angelopteromyia chvalai*). Acrophallic tubes in *Angelopteromyia korneyevi* sp. n. are equal in diameter and length (unequal (one thick and one thin) in *Angelopteromyia merzi*, and apically with cup-like extensions each in *Angelopteromyia alf*).

### Key to species of *Angelopteromyia*

**Table:** 

1	Basal and costal cells dark brown and without hyaline spots (fig. 1), face shining back, femur and tibia black, Fore femur ventro-laterally with 2 rows of slightly thickened long black setae. Male genitalia: acrophallic tubes equal in diameter and length, apex of proctiger triangular in profile (fig. 3)	*Angelopteromyia korneyevi* sp. n.
–	Basal and costal cells with several hyaline spots. Other characters variable	2
2	Lower half of face and femora shining black. Fore femur postero-ventrally with black non-thickened setae. Male genitalia: acrophallic tubes unequal (one thick and one thin), apex of proctiger rounded (Korneyev 2001: fig. 51)	*Angelopteromyia merzi*
–	Lower half of face at least in the middle densely microtrichose; femora more or less microtrichose. Fore femur postero-ventrally either with black thickened or with fine white setae. Other characters variable	3
3	Lower half of face below antennal grooves with 2 large shining black areas; facialia strongly projected antero-laterally, genal ridge well-developed (especially in females) (Korneyev 2001: figs 22–23); fore femur postero-ventrally with fine white setae. Anterior portion of mesonotum whitish setulose, with shining black anterior margin. Wing of female proximally of costagial break with large triangular projection of costal vein (Korneyev 2001: fig. 39). Abdomen whitish setulose. Male genitalia: Acrophallic tubes equally thick (Korneyev 2001: fig. 41), apex of proctiger rounded. Female abdominal pleura with bunch of long yellowish white setulae (Korneyev 2001: figs 43–44)	*Angelopteromyia alf*
–	Lower half of face below antennal grooves completely microtrichose or at most with two black dots; facialia not projected antero-laterally, genal ridge poorly developed in both sexes (Korneyev 2001: figs 24–25); fore femur postero-ventrally with thickened and short, spine-like black setae. Anterior portion of mesonotum black setulose, without shining black marginal area. Wing of female proximally of costagial break without any modification of costal vein. Abdomen black setulose. Male genitalia not examined. Female abdominal pleura velvet black, without long yellowish-white setulae	*Angelopteromyia chvalai*

## Supplementary Material

XML Treatment for Angelopteromyia korneyevi
